# Hepatitis B virus X protein promotes the stem-like properties of OV6^+^ cancer cells in hepatocellular carcinoma

**DOI:** 10.1038/cddis.2016.493

**Published:** 2017-01-19

**Authors:** Chao Wang, Ming-da Wang, Peng Cheng, Hai Huang, Wei Dong, Wei-wei Zhang, Peng-peng Li, Chuan Lin, Ze-ya Pan, Meng-chao Wu, Wei-ping Zhou

**Affiliations:** 1 The Third Department of Hepatic Surgery, Eastern Hepatobiliary Hospital, Second Military Medical University, 225 Changhai Road, Shanghai 200438, China; 2 Department of Urology, Changhai Hospital, Second Military Medical University, 168 Changhai Road, Shanghai 200438, China; 3 The Department of Hepatic Surgery, Eastern Hepatobiliary Hospital, Second Military Medical University, 225 Changhai Road, Shanghai 200438, China; 4 Changhai Hospital, Second Military Medical University, 168 Changhai Road, Shanghai 200438, China; 5 Changzheng Hospital, Second Military Medical University, Fengyang Road Shanghai 200003, China; 6 Data Scientist, Liberty Mutual Group, 157 Berkeley Street, Boston, MA 02116, USA

## Abstract

Hepatitis B virus X protein (HBx) and cancer stem-like cells (CSCs) have both been implicated in the occurrence and development of HBV-related hepatocellular carcinoma (HCC). However, whether HBx contributes to the stem-like properties of OV6^+^ CSCs in HCC remains elusive. In this study, we showed that the concomitant expression of HBx and OV6 was closely associated with the clinical outcomes and prognosis of patients with HBV-related HCC. HBx was required for the stem-like properties of OV6^+^ liver CSCs, including self-renewal, stem cell-associated gene expression, tumorigenicity and chemoresistance. Mechanistically, HBx enhanced expression of MDM2 by directly binding with MDM2 and inhibiting its ubiquitin-directed self-degradation. MDM2 translocation into the nucleus was also upregulated by HBx and resulted in enhanced transcriptional activity and expression of CXCL12 and CXCR4 independent of p53. This change in expression activated the Wnt/*β*-catenin pathway and promoted the stem-like properties of OV6^+^ liver CSCs. Furthermore, we observed that the expression of any two indicators from the HBx/MDM2/CXCR4/OV6 axis in HCC biopsies could predict the prognosis of patients with HBV-related HCC. Taken together, our findings indicate the functional role of HBx in regulating the stem-like properties of OV6^+^ CSCs in HCC through the MDM2/CXCL12/CXCR4/*β*-catenin signaling axis, and identify HBx, MDM2, CXCR4 and OV6 as a novel prognostic pathway and potential therapeutic targets for patients with HBV-related HCC patients.

Liver cancer is the second leading cause of cancer death in men worldwide.^1^ Hepatocellular carcinoma (HCC) is the most common type of primary liver cancer.^2^ Chronic infection with hepatitis B virus (HBV) is a major risk factor for HCC.^3^ Furthermore, hepatitis B virus X protein (HBx), encoded by HBV, is known to be necessary in the occurrence and development of HCC.^4^ Our group has demonstrated that HBx promotes the expansion and tumorigenicity of hepatic progenitor cells (HPCs), which contribute to HBx-mediated tumor formation in a DDC-induced mouse model.^5^ Accumulating evidence suggests that HBx modulates the transcriptional activities of many genes involved in cell survival and apoptosis by interacting with certain components of signal pathways such as Nrf2 and *β*-catenin.^6, 7^ In addition, HBx regulates the degradation of various proteins, including c-Myc, by interacting with components of the ubiquitin-proteasome system.^8^ However, the molecular mechanisms involved in the association of HBx with HBV-related hepatocarcinogenesis remain elusive.

Although surgical resection and liver transplantation are curative treatments for HCC patients, most HCCs are prone to invasion and metastasis, and the long-term prognosis remains unsatisfactory. For patients in the advanced stages of HCC, transarterial chemoembolization (TACE) is the second-line treatment, but its overall effect is less than satisfactory due to high chemoresistance of the tumor.^9^ Because of this, many reports have posited that cancer stem-like cells (CSCs) can be enriched by chemotherapeutic drugs, which is closely related to drug resistance and eventual relapse of HCC.^10, 11^ For instance, angiopoietin-like protein 1 (ANGPTL1) inhibits sorafenib resistance and cancer stemness in HCC cells by repressing EMT.^11^ Therefore, it is essential to thoroughly understand the molecular mechanism underlying CSCs in the progression of HCC to develop more effective therapeutic strategies.

The CSC theory proposes that CSCs are defined as a subpopulation of cells in tumors that share similarities with normal stem cells, such as self-renewing capacity. The existence of CSCs was first reported in acute myeloid leukemia^12^ and more recently in various solid tumors, including those of the brain, liver and breast.^13, 14, 15^ Recently, CSCs in HCC have been identified by cell surface antigens such as CD133, EpCAM, CD24 and CD90.^16, 17, 18, 19^ Previous studies conducted by our group and others also identified OV6 as a marker for CSCs in HCC and demonstrated that OV6^+^ cancer cells promoted HCC progression through the C-X-C chemokine receptor type 4 (CXCR4) and its specific ligand CXCL12 (SDF-1).^20^ The CXCR4/CXCL12 pathway has been implicated in the invasive and stemness properties of cancer.^21^ Upon CXCL12 binding, CXCR4 activates different pathways such as the PI3K/AKT and Wnt/*β*-catenin pathways, both of which contribute to the maintenance of the stem-like properties of CSCs.^22^ However, there is still a limited understanding of the molecular mechanisms underlying CSCs in the invasion and metastasis of HCC.

In the present study, we demonstrated that concomitant HBx and OV6 expression was closely correlated to the clinicopathological characteristics and prognosis of HBV-related HCC patients. Gene expression microarrays were utilized to demonstrate that MDM2 was the critical gene involved in HCC carcinogenesis. MDM2 is an oncogene originally identified as being amplified on double-minute chromosomes in transformed mouse fibroblasts.^23^ MDM2 has been shown to have an important role in cancers, stem cells and cancer stem cells.^24, 25, 26^ In addition, MDM2 is a member of the RING finger family of E3 ubiquitin ligases and negatively regulates the tumor suppressor protein p53.^27^ However, recent studies have shown that MDM2 also exerts p53-independent activities.^28^ In our model, MDM2 was shown to be necessary for HBx to regulate the stem-like characteristics of OV6^+^ liver CSCs in a p53-independent manner. Our findings also showed that the HBx/MDM2/CXCR4/OV6 axis could serve as a prognostic indicator for HBV-related HCC patients.

## Results

### Concomitant elevated expression of HBx and OV6 predicts a poor prognosis for patients with HBV-related HCC

**Figure 1:**
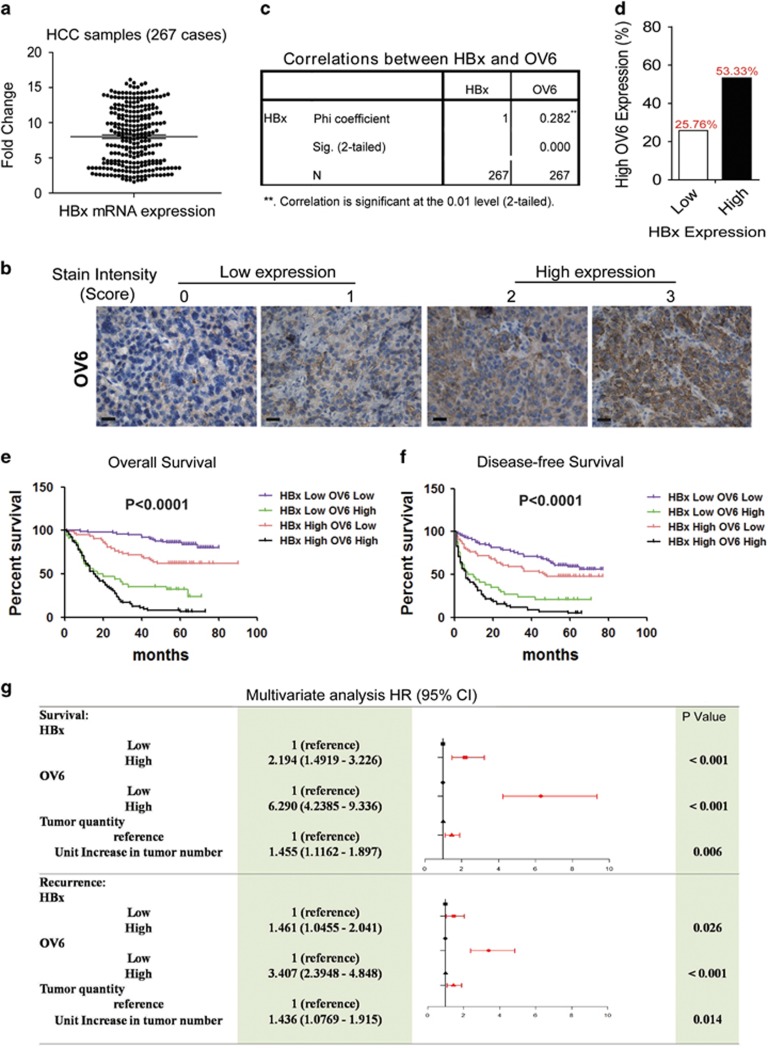
Concomitant elevated expression of HBx and OV6 predicts a poor prognosis for patients with HBV-related HCC. (**a**) The mRNA expression levels of HBx in 267 primary HCC specimens were evaluated by qRT-PCR. The mRNA levels of *β*-actin were used as an internal control. The expression level of HBx was determined using the delta-delta Ct method. (**b**) TMA sections of HCC were applied for immunohistochemical (IHC) staining for the expression of OV6 (*n*=267). The staining intensity of OV6 was scored from 0 to 3. Representative IHC images of OV6 staining in HCC specimens are shown (scale bar=50 *μ*m). (**c**) Correlation analysis of HBx and OV6 expression in HCC tissues (Chi coefficient=0.282; *P*<0.0001). (**d**) The percentage of patients with relatively high OV6 staining was much greater in the HBx-high group when compared with that in the HBx-low group (53.33 *versus* 25.76%). (**e** and **f**) The overall survival (OS) and disease-free survival (DFS) rates of 267 patients with HCC were compared among different groups. (**g**) Multivariate analysis of the hazard ratios (HRs) for overall survival and tumor recurrence. Univariate Cox proportional hazard (PH) analyses were conducted for each variable and covariate. A multivariate Cox PH regression model was established, and the HRs were presented as the means (95% confidence interval). Only variables that met the PH assumption in the univariate analysis would be included in the multivariate Cox PH analysis

Our previous results have demonstrated that HBx may promote the expansion and malignant transformation of HPCs, which contributes to HBx-driven tumorigenicity.^5^ In this study, we further investigated the potential role of HBx in regulating putative liver CSCs. Our study identified OV6 as a novel biomarker of the liver CSC subpopulation in HCCs.^20^ First, we determined the expression of HBx and OV6 in 267 patients with primary HCC using tissue microarrays (TMAs) (Figures 1a and b). A positive correlation between HBx and OV6 expression was observed in HBV-related HCC samples (Phi coefficient=0.282; *P*<0.0001; Figure 1c). Based on the expression of HBx and OV6, all 267 patients were divided into four groups: I (*n*=72), HBx^high^ and OV6^high^; II (*n*=63), HBx^high^ and OV6^low^; III (*n*=34), HBx^low^ and OV6^high^; and IV (*n*=98), HBx^low^ and OV6^low^ (Table 1). In patients with HBx^high^ HCC, the percentage with high OV6 staining was almost twice that of the HBx^low^ group (53.33% *versus* 25.76% Figure 1d). Moreover, compared with the other groups, the HBx^high^/OV6^high^ patients exhibited much more aggressive clinicopathological characteristics such as hepatitis B envelope antigen positivity (*P*=0.021), tumor size (*P*<0.001), tumor number (*P*=0.004) and microvascular invasion (*P*<0.001; Table 1). More importantly, the differences in both overall survival (OS) and disease-free survival (DFS) were significant among these four groups (Figures 1e and f). In addition, the multivariate analysis indicated that together with elevated OV6 levels and increased tumor numbers, upregulated HBx expression levels were an independent risk factor for both OS and DFS in HCC patients (Figure 1g). In conclusion, concomitant overexpression of HBx and OV6 can serve as an effective prognostic predictor for patients with HBV-related HCC.

### HBx enhances the stem cell-like properties and tumorigenic potential of OV6^+^ liver CSCs

**Figure 2:**
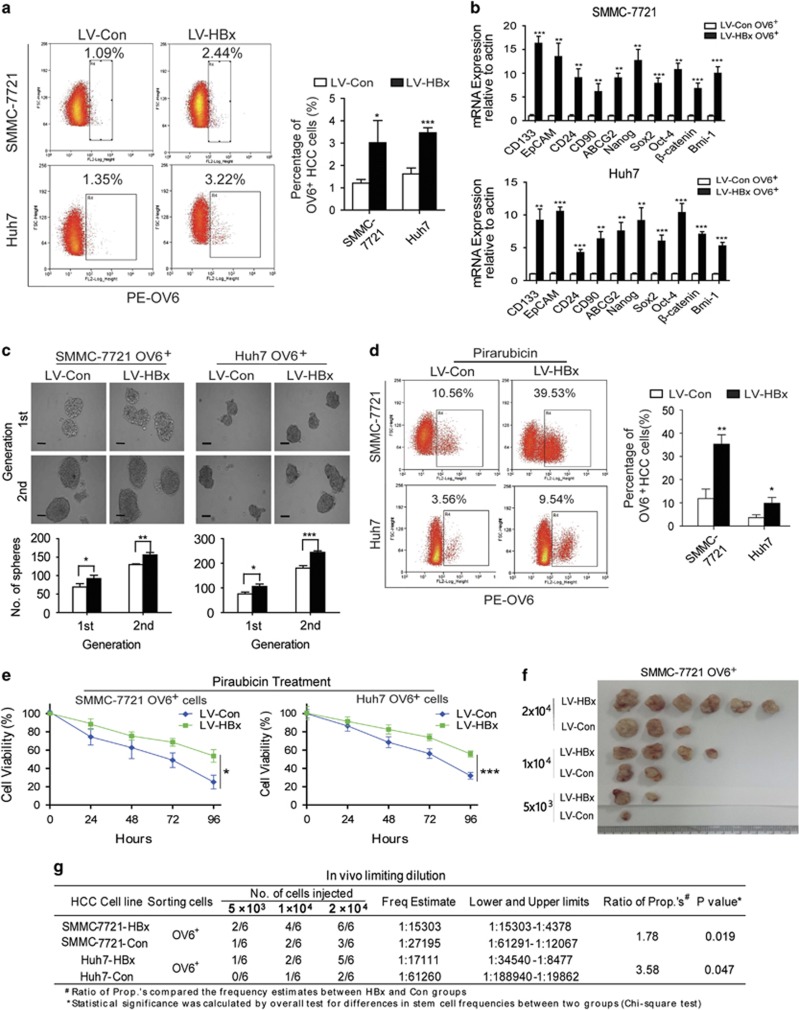
HBx enhances the stem cell-like properties and tumorigenic potential of OV6^+^ liver CSCs. (**a**) The percentage of OV6^+^ cells among the SMMC-7721 and Huh7 cells stably expressing either LV-HBx or LV-Con was measured via flow cytometry and presented as a dot plot analysis. Representative images are shown in the left panel. Experiments were performed in triplicate, and all data are shown as the mean±S.D. *P<0.05 and ***P<0.001. (**b**) The mRNA expression levels of multiple stemness-related genes in magnetically sorted LV-HBx OV6^+^ or LV-Con OV6^+^ cells from SMMC-7721 and Huh7 cultures were evaluated by qRT-PCR. The fold change was determined using the delta-delta Ct method. Quantified mRNA levels were normalized to *β*-actin and presented relative to the controls. The data are presented as the mean±S.D. **P<0.01 and ***P<0.001. (**c**) Representative images of primary and secondary passages of HCC spheres derived from magnetically sorted HCC-LV-HBx OV6^+^ and LV-Con OV6^+^ cells are shown (Scale bar=50 *μ*m; upper panel), and the number of tumor spheroids was counted (lower panel). Experiments were performed in triplicate, and results display the mean±S.D. *P<0.05, **P<0.01 and ***P<0.001. (**d**) HCC cells infected with either LV-HBx or LV-Con were treated with 5 *μ*M pirarubicin for 4 days, and the percentage of OV6^+^ cells were detected via flow cytometry. Representative results from three independent experiments are shown. (**e**) OV6^+^ HCC cells were treated with 5 *μ*M pirarubicin, and cell viability was measured by using the CCK-8 assay at the indicated time points. **P<0.05 and ***P<0.001. (**f** and **g**) Male NOD/SCID mice were subcutaneously injected with the indicated number of OV6^+^ HCC cells infected with either LV-HBx or LV-Con, and the tumor incidence in the mouse xenografts was evaluated. Representative subcutaneous xenograft tumors from mice injected with a gradient series of lentivirus-infected OV6^+^ cells after 6 weeks of transplantation are shown. The percentage of CSCs present was evaluated 6 weeks after injection by a limiting dilution assay

Next we explored whether overexpression of HBx could impact the stem-like characteristics of OV6^+^ liver CSCs. Using a lentivirus-based approach, we infected human HCC cells with lentivirus containing constructs expressing either HBx (LV-HBx) or empty vectors (LV-Con) and established HCC cells lines (SMMC-7721 and Huh7) that stably express HBx (Supplementary Figure S1). The flow cytometry results revealed that overexpression of HBx contributed to the expansion of the OV6^+^ population within the total HCC cell pool (Figure 2a). Moreover, OV6^+^ cells magnetically sorted from LV-HBx HCC cell lines, exhibited increased stemness-related genes (Figure 2b). Next we conducted *in vitro* spheroid formation assays to test whether HBx could regulate the self-renewal of liver CSCs. As expected, the OV6^+^ cells magnetically sorted from LV-HBx HCC cell lines were prone to form more tumor spheres when compared with OV6^+^ cells sorted from LV-Con HCC cell lines (Figure 2c). In addition, drug resistance is another important property of CSCs and may contribute to chemotherapeutic failure and tumor relapse.^29, 30^ To test whether HBx could manipulate the chemoresistance of liver CSCs, LV-HBx HCC cell lines were treated with chemotherapeutic agents commonly used in TACE treatment, including pirarubicin, oxaliplatin and hydroxycamptothecin (HCPT). The flow cytometry results revealed that HBx obviously enriched the chemo-resistant OV6^+^ subpopulation (Figure 2d and Supplementary Figure 2A) and enhanced the cell survival ability of these cells after exposure to anticancer drugs (Figure 2e and Supplementary Figure 2B), suggesting the critical role of HBx in regulating chemoresistance of OV6^+^ liver CSCs.

Next, we performed xenograft assays using a gradient series of OV6^+^ subpopulations sorted from either HCC-LV-HBx or HCC-LV-Con cells. As shown in Figures 2f and g, subcutaneous injection of HBx-expressing OV6^+^ HCC cells resulted in an increased incidence of tumorigenicity, indicating that HBx overexpression could enhance the tumorigenicity of OV6^+^ subpopulations *in vivo*. Taken together, our findings suggest that HBx is required for the expansion and maintenance of putative OV6^+^ liver CSCs.

### MDM2 is identified to be upregulated by HBx in OV6^+^ liver CSCs

**Figure 3:**
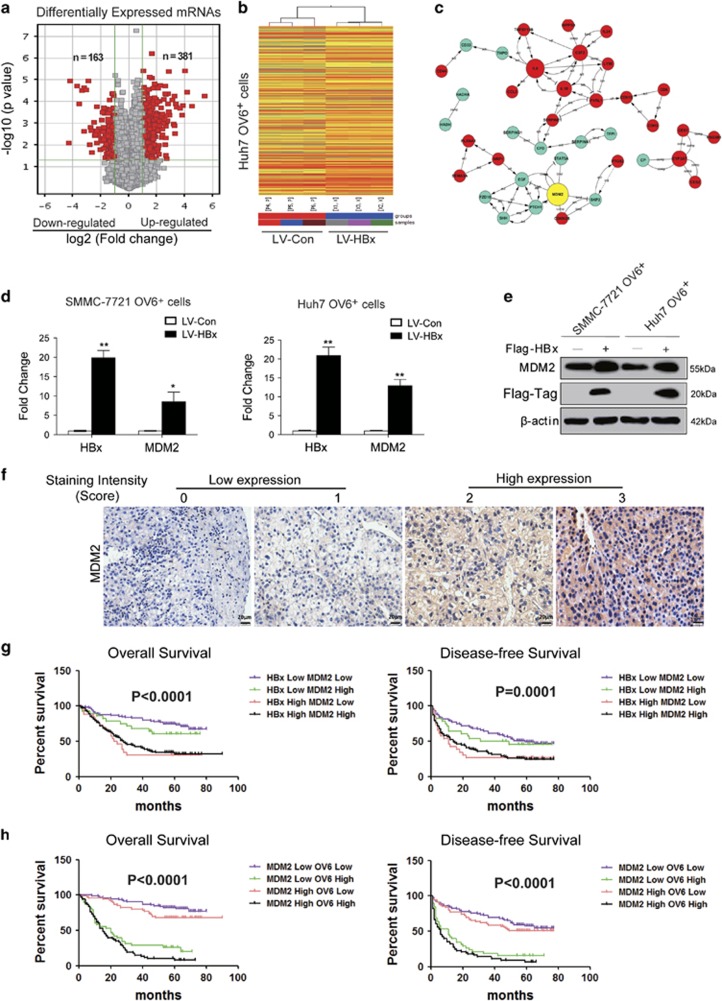
MDM2 is identified to be upregulated by HBx in OV6^+^ liver CSCs. (**a**) Gene expression was assessed in HCC cell samples using genome microarrays (Agilent Technologies; *n*=3 microarrays/group). A scatter-plot distribution of differentially expressed mRNA transcripts between two groups of samples (OV6^+^/LV-HBx *versus* OV6^+^/LV-Con cells) is shown. The values of the *x* axes represent the averaged normalized signal values (log2 scale). The green lines represent the fold change (the default fold-change value given is 2). The mRNAs marked ‘red' indicated a greater than 2-fold change of mRNA expression between the two compared groups of samples. (**b**) The heat map and hierarchical clustering show a distinguishable mRNA expression profiling among the samples. ‘Red' indicates high relative expression, and ‘blue' indicates low relative expression. X1–X3 represent samples of OV6^+^/LV-HBx Huh7 cells, and P4-P6 represent samples of OV6^+^/LV-Con cells. (**c**) The interaction network between the differentially expressed transcripts screened in the microarray experiments was drawn (red circles: upregulated genes; blue circles: downregulated genes). (**d**) The mRNA levels of MDM2 in OV6+ SMMC-7721 and Huh7 cells expressing empty vector and HBx were detected by qRT-PCR. The fold change was determined using the delta-delta Ct method. Quantified mRNA levels were normalized to β-actin and presented relative to the controls. The data are presented as the mean ± S.D. *P<0.05 and **P<0.01. (**e**) The protein expression levels of MDM2 in indicated OV6+ cells expressing empty vector and HBx were detected by western blotting. β-actin was used as internal loading control. (**f**) TMA sections of HCC specimens were subjected to IHC to determine the expression of MDM2 (*n*=267). Representative MDM2 expression levels in the HCC TMA sections from 267 patients are shown (scale bar=20 *μ*m). The staining intensity of MDM2 was scored from 0 to 3. (**g** and **h**) The overall survival (OS) and disease-free survival (DFS) rates of 267 HCC patients were compared among the four indicated different groups

To elucidate the underlying mechanisms by which HBx regulates the stem-like properties of OV6^+^ liver CSCs, we used gene expression microarrays to screen differentially expressed genes in OV6^+^/LV-HBx HCC cells compared with the levels in LV-Con-infected OV6^+^ cells. By using a 2-fold cut-off with a *P*-value <0.05, a total of 544 genes were identified to be differentially expressed between the OV6^+^ Huh7 cells expressing empty vector and cells expressing HBx (163 downregulated, 381 upregulated; Figure 3a). A hierarchical clustering heat map was used to show the distinguishable mRNA expression patterns among these samples (Figure 3b). We next performed Gene Ontology (GO) and pathway analyses to determine the functional roles of these differentially expressed genes in GO terms and biological pathways, respectively. As shown in Supplementary Figure S3A, most of the enriched GO terms for biological processes targeted by dysregulated genes were closely related to the inflammatory response, the DNA damage response, and cell proliferation. A detailed pathway analysis revealed the enrichment of signaling pathways mainly corresponding to cell adhesion, the p53 signaling pathway, and cytokine receptor interactions (Supplementary Figure S3B). We next explored the possible biological interactions of these differentially regulated genes using Ingenuity Pathway Analysis (IPA). The highest-rated signal transduction network among the dysregulated genes in OV6^+^/HBx-expressing cells was established. Intriguingly, the IPA analysis also identified MDM2 as the most highly scored upstream molecule in this network (Figure 3c). Mounting evidence has shown that MDM2 is frequently overexpressed in the majority of malignances and promotes tumor development by negatively regulating the p53 pathway.^31, 32, 33^ To substantiate our screening results, qRT-PCR and western blot assays were performed to verify the expression of MDM2 in sorted OV6^+^ cells expressing either HBx or the control vector. As expected, MDM2 were significantly upregulated in OV6^+^/LV-HBx HCC cells when compared with those in the control cells (Figures 3d and e). Next, we verified this correlation in clinical tissues and performed IHC to determine the expression levels of MDM2 in HCC TMAs (Figure 3f). As predicted, MDM2 expression was associated with HBx content in clinical HBV-related HCC biopsies (Phi coefficient=0.595; *P*<0.0001; Supplementary Figure S3C). Similarly, a significant correlation between MDM2 and OV6 expression was also observed (Phi coefficient=0.208; *P*=0.001; Supplementary Figure S3D). The clinical data further revealed that HBV-related HCC patients with elevated HBx and MDM2 levels exhibited a worse OS and a shorter disease-free survival period than other patients (Figure 3g and Supplementary Table S1). Meanwhile, concomitant overexpression of MDM2 and OV6 predicted rapid tumor relapse and a poor prognosis for HBV-related HCC patients (Figure 3h and Supplementary Table S2). Taken together, these data strongly suggest that HBx may promote the expansion of OV6^+^ liver CSCs in a MDM2-dependent manner.

### MDM2 is required for HBx to promote the stem-like properties of OV6^+^ liver CSCs

**Figure 4:**
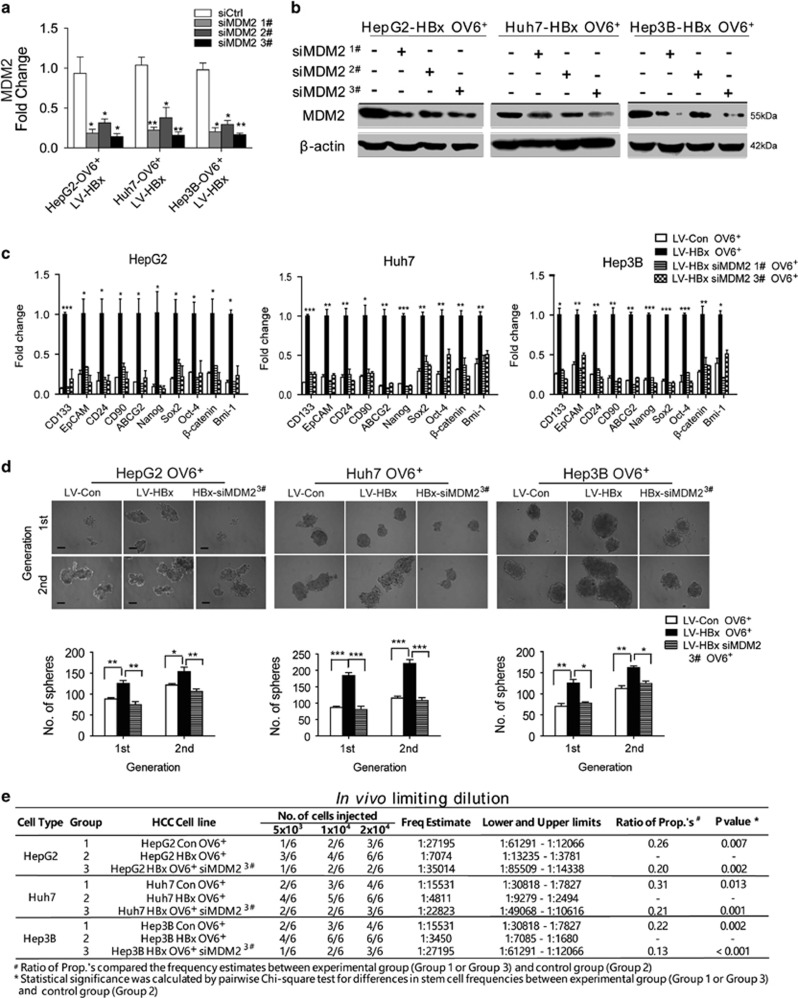
HBx mediates the self-renewal capacity and tumorigenicity of OV6^+^ liver CSCs via MDM2 independent of p53. (**a** and **b**) Isolated OV6^+^ HCC cells (HepG2, Huh7 and Hep3B) that stably expressed HBx were transfected with either negative control siRNAs (siCtrl) or siRNAs targeting MDM2 (three sequences: #1, #2, #3), and the mRNA and protein knockdown efficiency of MDM2 was detected by qRT-PCR and western blotting, respectively. The data in (**a**) are presented as the mean ± S.D. *P<0.05 and **P<0.01. (**c**) The mRNA expression levels of multiple stemness-related genes in OV6^+^ HBx-expressing cells transfected with siRNA targeting MDM2 (sequences: #1 and #3) were evaluated by qRT-PCR. The fold change was determined using the delta-delta Ct method. Quantified mRNA levels were normalized to *β*-actin and presented relative to the value observed in untransfected HBx-expressing cells. The data are presented as the mean±S.D. *P<0.05, **P<0.01 and ***P<0.001 compared with OV6^+^/LV-Con HCC cells. (**d**) Representative images of primary and secondary passages of HCC spheres derived from the indicated HCC cells are shown (scale bar=50 *μ*m; upper panel), and the number of tumor spheroids was counted (lower panel). All data are presented as the mean±S.D. *P<0.05, **P<0.01 and ***P<0.001. (**e**) NOD/SCID mice were subcutaneously injected with the different numbers of OV6^+^ LV-Con and LV-HBx cells transfected with either negative control siRNA or siMDM2 #3, and the tumor incidence in the mouse xenografts was evaluated. The percentage of CSCs was evaluated 6 weeks after injection by a limiting dilution assay

To further verify the essential role of MDM2 in the HBx-induced expansion of OV6^+^ liver CSCs, we knocked down MDM2 expression in LV-HBx-infected HCC cells using small interfering RNA (siRNA). As shown in Figures 4a and b, both sequence 1 and sequence 3 elicited a stable knockdown effect. MDM2 has been recognized as a negative regulator of the tumor suppressor p53, which is involved in tumorigenesis and progression of cancers.^31, 32^ Therefore, we investigated whether the involvement of MDM2 in maintaining the HBx-mediated stem-like properties of OV6^+^ CSCs was dependent on p53. Three HCC cell lines with different p53 expression states (HepG2, wild-type p53; Huh7, p53 mutant; and Hep3B, p53 deletion) were chosen for functional experiments. qRT-PCR analysis revealed that the mRNA levels of stemness-related genes were upregulated in sorted OV6^+^/LV-HBx-infected HCC cells, whereas MDM2 silencing resulted in significantly decreased expression of these genes regardless of the p53 status (Figure 4c). In addition, exogenous HBx expression enhanced the capacity of tumor sphere formation in OV6^+^ HCC cells, whereas downregulation of MDM2 abolished this effect (Figure 4d). Next, we assessed whether depleting MDM2 could impair the HBx-mediated tumorigenic potential of OV6^+^ CSCs *in vivo* by using a limiting dilution assay. Compared with OV6^+^ cells sorted from HCC cells transduced with LV-Con, an equivalent number of OV6^+^/LV-HBx HCC cells showed increased capability to form xenografts in NOD/SCID mice. Conversely, MDM2 knockdown decreased the tumorigenicity of OV6^+^/LV-HBx cells in our xenograft model (Figure 4e). Therefore, these data indicate that MDM2 promotes HBx-induced expansion of OV6^+^ liver CSCs in a p53-independent manner.

### HBx upregulates the expression of MDM2 by inhibiting its self-ubiquitination and degradation

**Figure 5:**
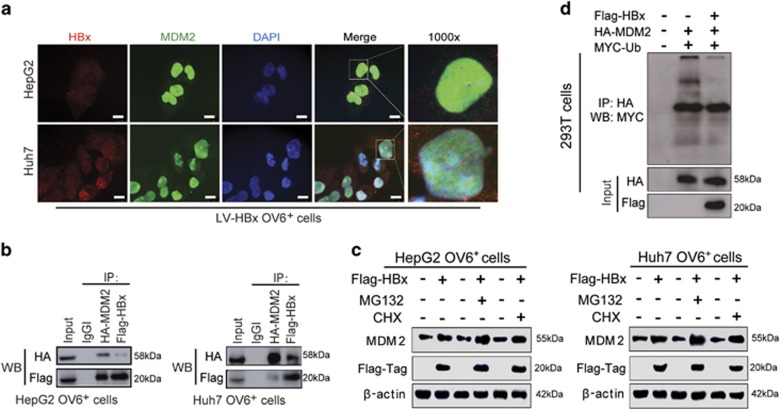
HBx upregulates the expression of MDM2 by inhibiting its self-ubiquitination and degradation. (**a**) OV6^+^ cells were magnetically sorted from HepG2 and Huh7 cells stably expressing HBx, followed by immunofluorescence assays. The localizations of Flag-tagged HBx (red) and MDM2 (green) were detected by confocal laser scanning microscopy. Nucleus were stained by DAPI dye (blue) and representative pictures are shown (scale bar=10 *μ*m). (**b**) Flag-HBx and HA-MDM2 were cotransfected into HCC (HepG2 and Huh7) cells and OV6^+^ cells were magnetically sorted from cell pool at 48 h after transfection. Whole-cell lysates were immunoprecipitated with agarose-conjugated anti-HA-tag or anti-Flag-tag antibody, or control immunoglobulin G, and analyzed by western blotting. (**c**) OV6^+^ HCC cells (HepG2 and Huh7) infected with either LV-HBx or LV-Con were treated with 1 *μ*g/ml cycloheximide (CHX) or 5 *μ*M MG132 for 12 h before collecting. Then cell lysates were subjected to western blotting. *β*-actin was used as internal loading control. (**d**) The ubiquitination of MDM2 was enhanced after HBx transfection for 48 h. Flag-HBx, HA-MDM2 and MYC-ubiquitin were cotransfected into 293T cells. At 48 h after transfection, whole-cell lysates were immunoprecipitated with agarose-conjugated anti-HA-tag antibody, and analyzed by western blotting with anti-HA-tag and anti-MYC-tag antibodies

Because MDM2 is required for HBx to promote the stem-like characteristics of OV6^+^ liver CSCs, we examined the mechanisms underlying HBx-mediated MDM2 activity. First, we determined whether HBx could directly interact with MDM2. An immunofluorescence staining assay showed the co-localization of HBx and MDM2 in the cytoplasm and nuclei of OV6^+^ HCC cells (Figure 5a), suggesting the direct interaction between HBx and MDM2. In addition, the co-immunoprecipitation assay revealed that HBx and MDM2 strongly interacted with each other in OV6^+^ CSCs sorted from HBx-expressing HCC cells (Figure 5b). Since MDM2 is an unstable protein that can be ubiquitinated in an autocatalytic manner, we next examined whether HBx inhibited the self-ubiquitination of MDM2. Western blotting was conducted to demonstrate that HBx-mediated upregulation of MDM2 was enhanced by MG132 (a proteasome inhibitor). Meanwhile, this upregulation was not blocked in the presence of cycloheximide (CHX), the protein synthesis inhibitor (Figure 5c). Therefore, these findings suggest that HBx regulated MDM2 degradation via a proteasomal-dependent pathway rather than by protein synthesis. As shown in Figure 5d, the ubiquitination of MDM2 was reduced when HBx was overexpressed in HCC cells. Taken together, HBx enhances expression of MDM2 by directly binding with MDM2 and inhibiting its self-ubiquitination and degradation activities.

### The CXCL12/CXCR4 autocrine loop is required for HBx/MDM2 to mediate the stem-like properties of OV6^+^ liver CSCs

**Figure 6:**
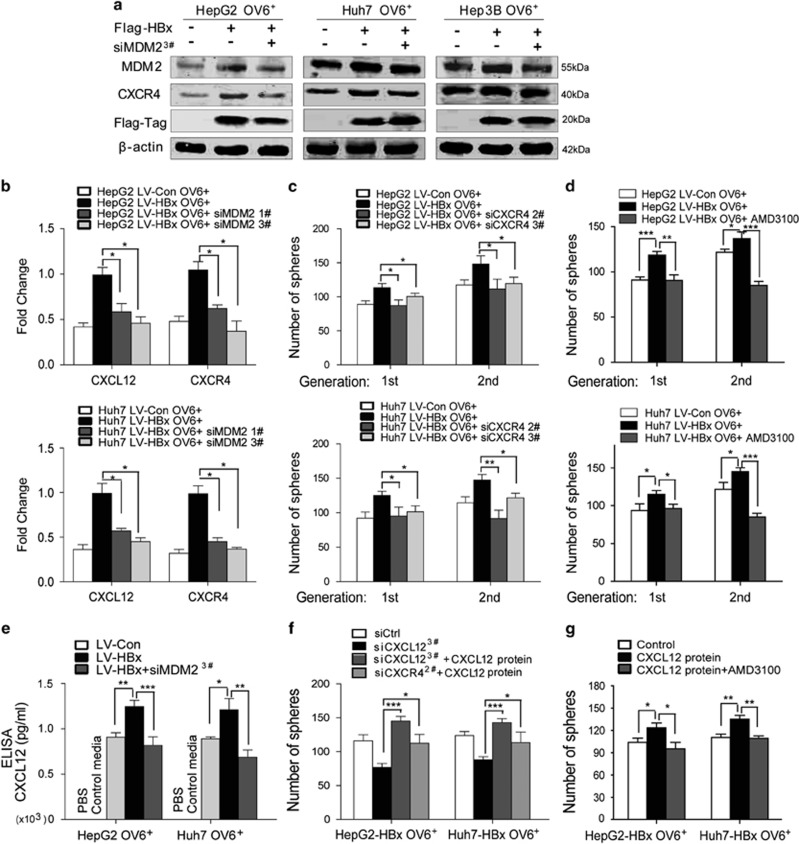
The CXCR4/CXCL12 autocrine loop is required in HBx/MDM2 mediation of the stem-like properties of OV6^+^ CSCs. (**a**) Western blotting was performed to assess the expression of MDM2, CXCR4 and Flag-HBx in OV6^+^ CSCs, OV6^+^ CSCs that overexpressed Flag-HBx and OV6^+^ CSCs that overexpressed Flag-HBx and MDM2 siRNA, which were magnetically isolated from HepG2, Huh7 and Hep3B cells, respectively. (**b**) Quantitative RT-PCR analysis was used to assess the CXCR4 and CXCL12 expression in OV6^+^ CSCs, OV6^+^ CSCs that overexpressed Flag-HBx and OV6^+^ CSCs that overexpressed Flag-HBx and MDM2 siRNA, which were isolated from HepG2 and Huh7 cells (*P<0.05). (**c** and **d**) Primary and secondary passages of HBx-expressing OV6^+^ CSCs from the HepG2 and Huh7 cell lines generated more spheroids. When MDM2 was knocked down in HBx-expressing OV6^+^ CSCs by siRNAs (siCXCR4, sequence #2 and #3) or AMD3100 treatment (20 *μ*g/ml), number of spheres was reduced. Experiments were performed in triplicate and all data represent mean±S.D., *P<0.05, **P<0.01 and ***P<0.001. (**e**) ELISA assay was performed to measure the CXCL12 protein level in PBS, media alone (DMEM) and conditioned media from OV6^+^ CSCs and HBx-expressing OV6^+^ CSCs transfected with either control siRNA or siRNA targeting MDM2 (*P<0.05, **P<0.01 and ***P<0.001). (**f**) The spheroid assay was used to assess number of spheres of HBx-expressing OV6^+^ CSCs transfected with control siRNA (siCtrl), siRNA targeting CXCL12 (sequence #3), or treated with recombinant CXCL12 protein plus siRNA targeting CXCL12, or recombinant CXCL12 protein plus siRNA targeting CXCR4. (**g**) The spheroid assay was conducted to assess the number of spheres of HBx-expressing OV6^+^ CSCs treated with recombinant CXCL12 protein alone, or recombinant CXCL12 protein plus AMD3100 at a concentration of 20 *μ*g/ml. In **f** and **g**, experiments were performed in triplicate. Representative results from three independent experiments are shown, and all data are presented as the mean±S.D., *P<0.05, **P<0.01 and ***P<0.001

Our previous study showed that activation of CXCR4 maintained the self-renewal capacity of OV6^+^ HCC cells.^20^ The CXCL12/CXCR4 pathway has been implicated in various types of cancers.^34^ Both the autocrine and paracrine effects of this pathway have been shown to maintain cancer growth and progression.^35^ Therefore, we investigated whether HBx/MDM2 regulated the stemness of OV6^+^ CSCs through the CXCL12/CXCR4 pathway. First, CXCR4 expression was upregulated by HBx in OV6^+^ CSCs in HCC, whereas MDM2 knockdown reversed HBx-mediated CXCR4 upregulation (Figures 6a and b). Subsequently, the siRNA approach was used to inhibit the expression of CXCR4 in HCC cells (Supplementary Figure S4A). The number of spheres was greater in HBx-expressing OV6^+^ CSCs compared with control OV6^+^ CSCs, whereas CXCR4 knockdown eliminated HBx-mediated self-renewal (Figure 6c). Similarly, inhibiting CXCR4 with its specific antagonist AMD3100 also attenuated the HBx-mediated self-renewal of OV6^+^ liver CSCs (Figure 6d). Furthermore, the qRT-PCR assay showed that CXCL12 expression was upregulated by HBx in OV6^+^ liver CSCs, but MDM2 depletion abolished this effect (Figure 6b). To examine the autocrine source of the CXCL12 ligand in cultured OV6^+^ CSCs, an ELISA assay was performed, which showed that CXCL12 protein was not found in control media (i.e, with no cells) compared with conditioned media from cultured OV6^+^ liver CSCs (Figure 6e). These data indicated that the *in vitro* source of CXCL12 protein is the OV6^+^ CSCs. Moreover, the protein levels of CXCL12 were increased in conditioned media from OV6^+^ CSCs overexpressing HBx and decreased in media from cultured OV6^+^ CSCs with HBx expression and MDM2 knockdown (Figure 6e). Furthermore, CXCL12 knockdown resulted in fewer spheres in HBx-expressing OV6^+^ CSCs, while the inhibited self-renewal was rescued by the addition of CXCL12 protein (Supplementary Figure S4B and Figure 6f). These data indicate that CXCL12 is secreted by OV6^+^ CSCs and is required in mediating the HBx/MDM2-induced stem-like properties of OV6^+^ liver CSCs. To further confirm that the effect of CXCL12 on the self-renewal of HBx-expressing OV6^+^ CSCs is dependent on CXCR4, we also added CXCL12 protein to the culture media of CXCR4-depleted HBx-expressing OV6^+^ CSC cells. As expected, we found no significant difference in the number of spheres after ligand stimulation compared with control cells (Figure 6f). However, inhibition of CXCR4 by AMD3100 significantly disrupted the effect of CXCL12 on the self-renewal capacity of HBx-expressing OV6^+^ CSCs (Figure 6g). In summary, these data demonstrate that the CXCL12/CXCR4 autocrine loop is critical in mediating the HBx/MDM2 induction of the stem-like properties of OV6^+^ liver CSCs.

### HBx/MDM2-induced upregulation of CXCL12/CXCR4 activates the Wnt/*β*-catenin pathway in OV6^+^ liver CSCs

**Figure 7:**
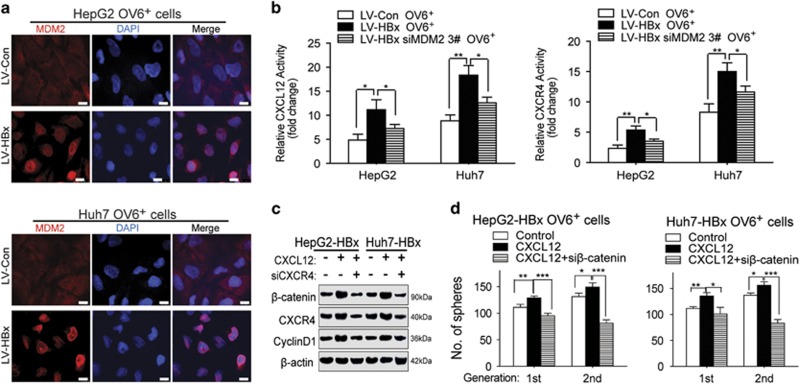
HBx/MDM2-induced upregulation of CXCL12/CXCR4 activates the Wnt/*β*-catenin pathway in OV6^+^ liver CSCs. (**a**) Immunofluorescence assays were performed using the OV6^+^ HCC cells (HepG2 and Huh7) infected with either LV-HBx or LV-Con. The intracellular localization of MDM2 (red) was detected by confocal laser scanning microscopy as indicated. Nucleus was stained by DAPI dye (blue; scale bar=10 *μ*m). (**b**) OV6^+^ LV-Con and LV-HBx HCC cells transfected with either negative control siRNA or siMDM2 were transfected with CXCL12 and CXCR4 luciferase reporters, followed by luciferase assay. The data display the mean±S.D., *P<0.05 and **P<0.01. (**c**) HBx-overexpressing HCC cells treated with control vehicle, recombinant CXCL12 protein alone or recombinant CXCL12 protein plus siRNA targeting CXCR4 for 48 h before collecting. The expression levels of indicated molecules in whole-cell lysates were detected by western blotting. *β*-actin was used as internal loading control. (**d**) The spheroid assay was performed to assess the number of spheres of HBx-expressing OV6^+^ CSCs treated with control vehicle, recombinant CXCL12 protein alone or recombinant CXCL12 protein plus siRNA targeting *β*-catenin. Experiments were performed in triplicate and all data represent the mean±S.D., *P<0.05, **P<0.01 and ***P<0.001

We next determined how HBx/MDM2 mediated CXCL12/CXCR4 expression. First, immunofluorescence staining showed that overexpression of HBx increased the amount of MDM2 localized in the nuclei of OV6^+^ liver CSCs (Figure 7a). Second, overexpression of HBx was demonstrated to enhance the transcriptional activation of CXCL12 and CXCR4 via luciferase reporter assays, whereas knockdown of MDM2 prevented HBx-induced transcriptional upregulation (Figure 7b). Our previous study demonstrated that the CXCL12/CXCR4 axis promoted self-renewal and the migration of OV6^+^ cells in HCC. In addition, *β*-catenin has a vital role in the maintenance of OV6^+^ liver CSCs. Therefore, we asked whether CXCL12/CXCR4 enhanced the stem-like properties of OV6^+^ liver CSCs by activating the Wnt/*β*-catenin pathway. First, western blotting showed that CXCL12 enhanced the expression of *β*-catenin and cyclin D1, whereas CXCR4 depletion blocked the ability of CXCL12 to activate the Wnt/*β*-catenin pathway in HBx-expressing OV6^+^ liver CSCs (Figure 7c). Furthermore, the spheroid formation assay was performed to demonstrate that the number of spheres from HBx-expressing OV6^+^ liver CSCs treated with CXCL12 was greater than that of untreated HBx-expressing OV6^+^ CSCs. However, CXCR4 knockdown attenuated the effect of CXCL12 on enhancing tumor spheroid formation in HBx-expressing OV6^+^ CSCs (Figure 7d). Taken together, HBx enhances the transcriptional activation of CXCL12 and CXCR4, which activate the Wnt/*β*-catenin pathway in OV6^+^ liver CSCs.

### HBx/MDM2/CXCR4/OV6 expression predicts malignant clinicopathological features and a poor prognosis for patients with HBV-related HCC

**Figure 8:**
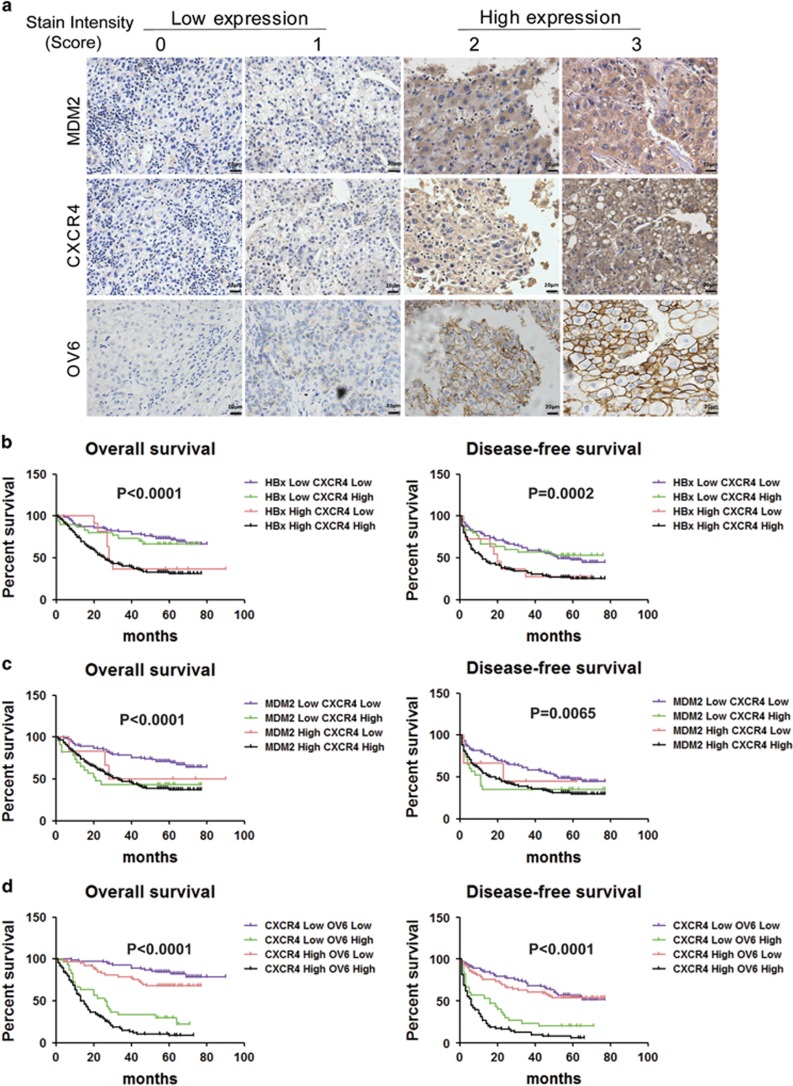
HBx/MDM2/CXCR4/OV6 expression predicts malignant clinicopathological features and a poor prognosis for patients with HBV-related HCC. (**a**) Representative IHC staining of MDM2, CXCR4 and OV6 in HBV-related HCC specimens are shown (scale bar=20 *μ*m). Kaplan–Meier curves for overall survival (OS) and disease-free survival (DFS) were compared according to combinations of HBx and CXCR4 (**b**), MDM2 and CXCR4 (**c**), and CXCR4 and OV6 expression (**d**) to assess their prognostic significance in HCC patients (*n*=267)

We also examined the clinical relationship among HBx/MDM2/CXCR4/OV6 expression in specimens from patients with HBV-related HCC. First, qRT-PCR and IHC analysis were conducted in HCC specimens from 267 patients (Figure 8a), which revealed a close correlation between HBx and CXCR4, MDM2 and CXCR4, and CXCR4 and OV6 (*P*<0.0001) (Supplementary Figures S5A–C). Second, we examined whether any two indicators among HBx/MDM2/CXCR4/OV6 could predict malignant clinicopathological features as well as the prognosis of HCC patients. On the basis of their expression levels in the tumors, we found that the presence of any two indicators among HBx/MDM2/CXCR4/OV6 could predict malignant clinicopathological features and a poor prognosis for patients with HBV-related HCC (Figures 8b–d and Supplementary Tables S1–3). These results suggest that a combination of any two parameters among HBx/MDM2/CXCR4/OV6 could serve as a powerful predictor for poor prognosis, further supporting the critical role of HBx/MDM2/CXCR4 signaling in OV6^+^ cancer cells in the progression of HCC.

## Discussion

Hepatocellular carcinoma (HCC) comprises the majority of liver cancer cases, and the high HCC rates in China largely reflect the elevated prevalence of chronic hepatitis B virus (HBV) infection.^36^ HBx has been demonstrated to have crucial roles in the relapse and metastasis of HCC.^37^ Our previous study proved that HBx could promote the expansion and tumorigenicity of HPCs in DDC-treated mice. Importantly, we also observed there was a positive correlation between HBx and OV6 expression in samples from patients with HBV-related HCC.^5^ These findings suggest that HBx may regulate the stem-like properties of OV6^+^ CSCs in HBV-related HCC. Consistent with this, other studies have reported that HBx contributes to hepatocarcinogenesis partially by promoting changes in gene expression that are characteristic of CSCs.^38, 39^ However, the molecular mechanisms underlying how HBx regulates the stem-like properties of CSCs remain elusive.

In this study, we demonstrated that HBx mediated the stem-like characteristics of OV6^+^ CSCs in HCC. A gene expression microarray demonstrated that MDM2 is upregulated in various types of tumors and especially increased in our model. Mounting evidence has demonstrated that MDM2 is a key negative regulator of the p53 tumor suppressor protein, which promotes tumor growth.^40^ In addition to p53, a number of additional interaction partners for MDM2 have been described.^41^ In our study, we demonstrated that HBx promoted the stem-like properties of OV6^+^ CSCs via MDM2 independent of p53. Other studies demonstrated that MDM2 had a crucial role in the stemness of CSCs from breast cancer and glioblastoma.^42, 43^ It is well established that MDM2 is an unstable member of the RING finger family of E3 ubiquitin ligases that is ubiquitinated in an autocatalytic manner. Our results demonstrated that HBx enhanced the expression of MDM2 by directly binding to MDM2 and inhibiting its self-degradation.

Subsequently, we presented evidence that HBx promoted increased translocation of MDM2 into the nucleus, which enhanced the transcriptional activation and expression of CXCL12 and CXCR4 in OV6^+^ liver CSCs. Because our previous study indicated that CXCL12/CXCR4 was necessary to maintain the stemness of OV6^+^ CSCs in HCC,^20^ we next assessed whether HBx/MDM2 regulated CXCL12/CXCR4 signaling. As expected, our data indicate that the CXCL12/CXCR4 autocrine loop is critical in HBx/MDM2 mediating the stem-like properties of OV6^+^ CSCs. We also proved that HBx/MDM2 regulation of CXCL12/CXCR4 signaling resulted in activation of the Wnt/*β*-catenin pathway in OV6^+^ liver CSCs.

In conclusion, HBx induces greater stem-like features of OV6^+^ CSCs in HCC via the MDM2/CXCL12/CXCR4/*β*-catenin signaling axis independent of p53. Furthermore, increased expression of any two indicators among HBX/MDM2/CXCR4/OV6 could predict the clinicopathological characteristics and prognosis of patients with HBV-related HCC. The related molecular mechanism will be discussed in depth in our future work.

## Materials and methods

### Clinical HCC tissue microassays

Ethic approval of this study was obtained from the Ethic Committee Board of Eastern Hepatobiliary Surgery Hospital (Shanghai, China), and informed consent was obtained from each patient. The HCC tissue microarrays containing 267 patients who underwent curative hepatectomy for HCC at our hospital were constructed and analyzed according to previous instructions.^44^ The clinicopathological features of patients were summarized in Supplementary Table S4. All the patients were followed until September 2013, with a median observation time of 54 months. Overall survival (OS) represented the interval between the date of surgery and death. Disease-free survival (DFS) was defined as the interval between the dates of surgery and recurrence.

### Cell lines and cell culture

HCC cell lines SMMC-7721, Huh7, HepG2, Hep3B and 293T cells were purchased from Cell Bank of Type Culture Collection of the Chinese Academy of Sciences (Shanghai, China). HCC Cell lines were routinely cultured in Dulbecco's modified Eagle's medium (Gibco, Carlsbad, CA, USA) supplemented with 10% fetal bovine serum (FBS, Gibco). Spheres from OV6^+^ HCC cells were cultured in DMEM/F-12 (Gibco) without FBS, but with supplement of ITS Liquid Media (Sigma-Aldrich, St. Louis, MO, USA), B27 (Invitrogen, Carlsbad, CA, USA), EGF Recombinant Human Protein Solution (Gibco), FGF-Basic (AA 1-156). Recombinant CXCL12 protein (R&D Systems, Minneapolis, MN, USA) was added in conditional media for the CXCL12/CXCR4 related assays. AMD3100 (Plerixafor, HY-10046) was purchased from MedChem Express (Monmouth Junction, NJ, USA). MG132 (M8699) and CHX (R750107) were obtained from Sigma-Aldrich.

### Lentivirus, and small interfering RNA

Human HBx gene was encoded in pLentiVX-EF1A vector (Intergrated biotech solutions, Co., Ltd, Shanghai, China) and the constructed lentiviral vectors were designated as lentiviral vector (LV)-HBx or LV-Con. Then the lentivirus containing constructs were transfected into HCC cells (SMMC-7721 and Huh7 cell lines) with a multiplicity of infection (MOI) 20 in the presence of Polybrene (Sigma-Aldrich; 8 *μ*g/ml). At 12 h after transfection, the original culture media was replaced with fresh media. After incubating for 48 h, cells were maintained in culture media with 1.5 *μ*g/ml of Puromycin (Sigma-Aldrich) to select stable HBx-expressing HCC cells. The small interfering RNAs (siRNAs) targeting MDM2, CXCR4, CXCL12, *β*-catenin (three sequences, defined as #1~#3; Supplementary Table S5) and negative control (scrambled siRNA) were purchased from Intergrated Biotech Solutions, Co., Ltd, Shanghai, China. The transient transfection of siRNAs was performed with Lipofectamine 2000 reagents (Invitrogen) according to the manufacturer's instructions. The supernatant was replaced with fresh medium containing 10% FBS 12 h post transfection.

### Quantitative real-time PCR

Total RNA from HCC cell lines and human HCC specimens were extracted using TRIzol reagent (Gibco) based on the manufacturer's protocols. Quantitative RT-PCR assays were carried out using an ABI 7300 Fast Real-Time PCR System (Applied Biosystems, Carlsbad, CA, USA) and SYBR Green PCR kit (Applied TaKaRa, Shiga, Japan). The primer sequences listed in Supplementary Table S6 were designed and purchased from Intergrated Biotech Solutions, Co., Ltd. Each measurement was performed in triplicate and the results were normalized by the expression of the *β*-actin gene. Fold change relative to mean value was determined by 2^−ΔΔ^.

### Western blotting and co-immunoprecipitation

Whole-cell extracts were prepared in lysis buffer and western blotting assays were performed as described previously.^45^ Briefly, cultured cells or liver cancer tissues were lysed in Triton lysis buffer (20 mM Tris, pH 7.4, 137 mM NaCl, 10% glycerol, 1% Triton X-100, 2 mM EDTA, 1 mM PMSF, 10 mM sodium fluoride, 5 mg/ml of aprotinin, 20 mM leupeptin and 1 mM sodium orthovanadate) and centrifuged at 12 000 × *g* for 15 min. Protein concentrations were determined via the BCA assay kit according to the manufacturer's protocols. Specific primary antibodies were shown as following: anti-MDM2 and anti-*β*-actin (Santa Cruz Biotechnology, Dallas, TX, USA), anti-CXCR4 (Abcam, Cambridge, MA, USA), anti-Flag-tag, anti-HA-tag, anti-MYC-tag, cyclin D1 and anti-*β*-catenin (Cell Signaling Technology, Danvers, MA, USA). The immunocomplexes were incubated with the fluorescein conjugated secondary antibody and then analyzed using an Odyssey fluorescence scanner and LI-COR imaging system (Li-COR Biosciences, Lincoln, NE, USA). For immunoprecipitation experiments, a total of 1 mg of HCC cell lysates were incubated with 2 *μ*g anti-Flag, or 2 *μ*g anti-HA antibodies or normal rabbit immunoglobulin G (Santa Cruz Biotechnology) for 8 h at 4 °C, followed by addition of Protein A/G Plus-Agarose (Santa Cruz Biotechnology) for another 3 h. The samples were denatured and subjected to western blotting analyses.

### *In vitro* ubiquitination analysis

To determine ubiquitin-conjugated endogenous MDM2, HA-MDM2 and MYC-Ub plus Flag-HBx plasmids were cotransfected into 293T cells using jetPEI (Polyplus, New York, NY, USA) for 48 h before collecting. Whole-cell extracts were prepared in lysis buffer, as mentioned above. The cell lysates were incubated with HA-conjugated beads (Invitrogen) overnight at 4 °C. Immunoprecipitated proteins were analyzed by western blotting using an anti-MYC antibody.

### Immunofluorescence staining

The indicated OV6^+^ HCC cells were seeded onto coverslips in 12-well plates and stained with rabbit anti-Flag (Cell Signaling Technology), anti-MDM2 (Santa Cruz Biotechnology) or mouse anti-MDM2 (Abcam) primary antibodies, followed by fluorescent staining with Alexa Fluor 555-conjugated IgG and Alexa Fluor 488-conjugated IgG (Invitrogen). Nuclear staining was conducted by 4,6-diamidino-2-phenylindole (DAPI). Representative images were captured with a Leica confocal microscope (Leica, Mannheim, Germany).

### Immunohistochemistry

HCC tissue microarray slides were stained with the following primary antibodies at 4 °C overnight: anti-OV6 (R&D Systems), anti-MDM2 (Santa Cruz Biotechnology) and anti-CXCR4 (Abcam). Corresponding secondary antibodies and diaminobenzidine (DAB) reagent (Dako, Carpinteria, CA, USA) were used in the detection procedure. All TMA slides were observed and photographed with an Olympus microscope (IX-70 OLYMPUS, Shinjuku, Tokyo, Japan). The staining levels of OV6, MDM2 and CXCR4 in all clinical samples were examined by two independent observers as described previously.^20^ The staining intensities were scored as 0 (negative), 1 (weakly positive), 2 (moderately positive) or 3 (strongly positive). Low expression level was defined as score ⩽1, whereas high expression in tumoral tissues was identified as score ⩾2.

### Flow cytometry and magnetic cell sorting

The magnetic-activated cell sorting and flow cytometric assay were carried out by using a Moflo XDP Cell Sorter (Beckman, Boulevard Brea, CA, USA). HCC cell lines were magnetically labeled with PE-conjugated-OV6 antibody (mouse IgG1; Miltenyi Biotec, Auburn, CA, USA) and subsequently incubated with rat anti-mouse IgG1 micro-Beads, and then separated on MACS MS column (Miltenyi Biotec). All the procedures were performed according to the manufacturer's instructions. HCC cells infected with LV-Con or LV-HBx were incubated with OV6 antibody and the percentages of OV6^+^ cells were measured by flow cytometry. For chemotherapy resistance experiments, HCC cells that stably expressed vector or HBx were treated with pirarubicin, oxaliplatin or HCPT for 48 h and the proportions of OV6^+^ cells were quantified using flow cytometry as mentioned above. For cell sorting experiments, OV6^+^ cell populations were magnetically sorted from HCC cells that stably expressed HBx or empty vectors and then subjected to *in vitro* functional studies or *in vivo* tumorigenicity experiments.

### Spheroid formation assay

For HCC cell lines, single-cell suspensions of 3000 magnetic sorted OV6^+^ cells were maintained in six-well ultra-low attachment culture microplates (Corning, Steuben County, NY, USA) for 14 days. For the second generation of sphere formation, formed spheroids were re-trypsinized to single-cell suspensions and equivalent number of isolated OV6^+^ cells were re-suspended in low attachment cultural conditions for another 14 days. The spheroid number was counted under a microscopy 14 days after seeding and the representative pictures were taken.

### Cell viability assay

The cell viability assay was performed with cholecystokinin-8 (CCK-8; Dojindo, Japan) as previously reported.^46^ Briefly, 6000 viable cells were seeded in triplicates in 96-well plates and treated with pirarubicin, oxaliplatin or HCPT since the onset of culture period for 4 days. At indicated time points, each well was added with 10 *μ*l CCK-8 and incubated for another 1 h. Then, the OD values were determined via a microplate reader (Synergy HT, BioTek, Winooski, VT, USA) at an absorbance of 450 nm. The cell viability rates were presented as a percentage of the control value which was recorded at the beginning (Time 0).

### Luciferase reporter assay

A dual-luciferase reporter assay was performed by using wild-type TCF-luciferase construct (pGL3-OT) and the mutant TCF-luciferase reporter construct (pGL3-OF). The promoter region of CXCR4 and CXCL12 were amplified by PCR. The pGL3-CXCR4 or CXCL12-luciferase plasmids were constructed by inserting these PCR products into the pGL3-Report Dual-Luciferase reporter vector (Ribobio Co., Guangzhou, China). Indicated HCC cells were cultured in 24-well plates. After incubating for 24 h, each well was transfected by lipofectamine 2000 (Invitrogen) with 500ng/well CXCR4 or CXCL12-luciferase plasmids with jetPEI reagents (Polyplus). Meanwhile, all wells were cotransfected with pRL-TK plasmids which contained the Renilla luciferase genes as internal control for normalizing transfection efficiency. Then the luciferase activity was determined using the Promega luciferase assay reagent and Synergy 2 Multi-Detection Microplate Reader after 48 h of incubation. The relative transcriptional activities of CXCL12 and CXCR4 were presented as the OT/OF ratio.^47^

### Microarray analysis of gene expression

The gene expression profiles were determined by Agilent human cDNA Microarray assay (Agilent Technologies, Santa Clara, CA, USA). Total RNA was extracted from OV6^+^/LV-Con and OV6^+^/LV-HBx Huh7 cells expressing using an RNeasy Mini kit (Qiagen, Hilden, Germany). Then, 10 *μ*g of total RNA was reverse-transcribed to produce biotin-labeled cRNA and fragmented by adding Blocking Agent and Fragmentation Buffer. Then the hybridization solution was dispensed into the gasket slide and assembled to the mRNA expression microarray slide. The slides were incubated for 17 h at 65 °C in an Agilent Hybridization Oven. The hybridized arrays were washed, fixed and scanned with using the Agilent DNA Microarray Scanner (part number G2505C). Agilent Feature Extraction software (version 11.0.1.1) was used to analyze acquired array images. Quantile normalization and subsequent data processing were performed with using the GeneSpring GX v11.5.1 software package (Agilent Technologies). After quantile normalization of the raw data, identified mRNAs were chosen for further data analysis. The detected signals were evaluated by gene hierarchical clustering of logarithmic values. Cluster 3.0 and Java TreeView 1.0.13 software were applied to generate a dendrogram for each cluster of genes according to their expression profiles.

### GO and ingenuity pathway analysis

The GO analysis functionally assessed the differentially expressed genes with GO categories which were derived from Gene Ontology. The ontologic pathway enrichment analyses for the differently expressed genes were performed with particular attention to GO biological processes. *P*-values less than 0.05 were considered significant. Pathway analysis was applied to identify the significant pathways based on the KEGG database. The inter-regulation network analysis was carried out by BioGenius Co. (Shanghai, China). For ingenuity pathway analysis (IPA), the identified genes with altered expression profiles from array assays were imported into the IPA Tool (Ingenuity H Systems, Redwood City, CA, USA). Then the biological networks, global functions and functional pathways of a specific data set were recognized by the IPA analyses.

### *In vivo* xenograft assay

Six-week-old male nonobese diabetic/severe combined immunodeficient (NOD/SCID) mice were purchased from Chinese Science Academy (Shanghai, China). All animal experiments were carried out according to the Second Military Medical University Animal Care Facility and the National Institutes of Health guidelines. OV6^+^ HCC cells expressing vector or HBx were subcutaneously injected into right flank of mice at different cell numbers as indicated. The proportion of OV6^+^ CSCs was evaluated by an *in vivo* limiting dilution assay according to the manufacturer's instructions.

### ELISA assay

Human CXCL12/SDF-1 ELISA Kit (BioVision, Milpitas, CA, USA) was applied in our ELISA assays. All procedures are in accordance with the manufacturer's instructions. Briefly, bring all the reagents and samples to room temperature before use. Add 100 *μ*l of each standard and sample into appropriate wells. Cover well and incubate for 2.5 h at room temperature or overnight at 4 °C with gentle shaking. Discard the solution and wash four times with 1 × Wash Solution. Add 100 *μ*l of 1 × prepared biotinylated antibody to each well. Incubate for 1 h at room temperature with gentle shaking. Discard the solution. Repeat the wash steps. Add 100 *μ*l of prepared Streptavidin solution to each well. Incubate for 45 min at room temperature with gentle shaking. Discard the solution. Repeat the wash steps. Add 100 *μ*l of TMB One-Step Substrate Reagent to each well. Incubate for 30 min at room temperature in the dark with gentle shaking. Add 50 *μ*l of Stop Solution to each well. Read at 450 nm immediately and finally calculate the related value.

### Statistical analysis

This work was supported by National Natural Science Foundation of China (81301861); National Natural Science Foundation of China (81672352); Shanghai Natural Science Foundation of China (13ZR1450700). State Key Infection Disease Project of China (2012ZX10002010); Shanghai New Excellent youth plan (XYQ2013074); National Key Basic Research Program of China (2014CB542102); Science Fund for Creative Research Groups, NSFC, China (81521091); the National High Technology Research and Development Program of China (2013AA032202); National Natural Science Foundation of China (81372207).

## Figures and Tables

**Table 1:** Clinicopathologic characteristics of HCC subtypes defined by OV6 and HBx expression

	**OV6/HBx expression**				
		**Either high**			
**HCC subtypes**	**Both high (*n*=72)**	**High HBx, low OV6 (*n*=63)**	**High OV6, low HBx (*n*=34)**	**Both low (*n*=98)**	***P*-value**^a^
*Sex*					
Male	63	54	28	91	0.307
Female	9	9	6	7	
					
Age (year)^b^	51.6±1.2	49.6±1.3	48.6±1.9	50.4±1	0.487
					
*HBeAg positive*					
Yes	25	10	4	20	0.021
No	47	53	30	78	
					
*AFP (ng/ml)*					
⩾ 400	54	34	20	59	0.066
< 400	18	29	14	39	
					
Tumor size (cm)^b^	7.6±0.5	5.5±0.4	7.4±0.7	4.9±0.3	<0.001
Tumor number (count)^b^	1.3±0.1	1.2±0.1	1.4±0.1	1.1±0.03	0.004
					
*Tumor differentiation grade*					
Well (I)	0	0	1	2	0.369
Intermediate (II–III)	72	63	33	96	
					
*Tumor satellites*					
Yes	49	45	28	69	0.493
No	23	18	6	29	
					
*Microvascular Invasion*					
Yes	51	45	23	44	<0.001
No	21	18	11	54	
					
*Recurrence*					
Yes	66	33	27	41	<0.001
No	6	30	7	57	
					
*Expired*					
Yes	67	24	24	16	<0.001
No	5	39	10	82	
					
Risk-free survival time (mo)^b^	12.1±1.8	38.5±3.2	20.1±3.9	47.7±2.2	<0.001
Overall survival time (mo)^b^	20.3±2	48.8±2.7	29.1±4.2	59.4±1.3	<0.001

a Statistical significance was caluculated by chi-square test or Fisher's exact test for categorical/binary measures and ANOVA for continuous measures.

b Data are presented as mean±S.D.
