# Supplementary material for: Hepatitis B virus X protein promotes the stem-like properties of OV6+ cancer cells in hepatocellular carcinoma
Source: Cell Death Dis. 2017 Jan 19;8(1):e2560–. doi: 10.1038/cddis.2016.493 (PMC5386392; doi:10.1038/cddis.2016.493)
Supplement: Supplementary Information [file cddis2016493x1.docx]

**Hepatitis B virus X protein (HBx) promotes the stem-like properties of OV6^+^ cancer cells in hepatocellular carcinoma**

Chao Wang*^1,2^, Ming-da Wang*^3^, Peng Cheng*^4^, Hai Huang^5^, Wei Dong^6^, Wei-wei Zhang^4^, Peng-peng Li^1^, Chuan Lin^1^, Ze-ya Pan^1^, Meng-chao Wu^3^ and Wei-ping Zhou**^#^**^1^.

**Author affiliations**

1. The Third Department of Hepatic Surgery, Eastern Hepatobiliary Hospital, Second Military Medical University, 225 Changhai Road, Shanghai 200438, China.

2. Department of Urology, Changhai Hospital, Second Military Medical University, 168 Changhai Road, Shanghai 200438, China.

3. The Department of Hepatic Surgery, Eastern Hepatobiliary Hospital, Second Military Medical University, 225 Changhai Road, Shanghai 200438, China.

4. Changhai Hospital, Second Military Medical University, 168 Changhai Road, Shanghai 200438, China.

5. Changzheng Hospital, Second Military Medical University, Fengyang Road, Shanghai 200003, China.

6. Data Scientist, Liberty Mutual Group, 157 Berkeley Street, Boston, MA02116.

* These authors contributed equally to the study.

**Supplementary Tables**

**Table S1. Clinicopathological Characteristics of HCC Subtypes Defined by MDM2 and HBx Expression.**

| **Clinicopathological features of HCC Subtypes Defined by MDM2 and HBx expression** | | | | | | | | | | | |
| --- | --- | --- | --- | --- | --- | --- | --- | --- | --- | --- | --- |
|  | **MDM2/HBx expression** | | | | | | | | |  | |
|  |  | | | **Either high** | | |  | | |  | |
| **HCC subtypes** | **Both high (n= 109)** | | | **High MDM2, low HBx (n= 28)** | **High HBx, low MDM2 (n= 26)** | | **Both low (n= 104)** | | | **P value^a^** | |
| **Sex** | |  |  | | |  | |  |  | |  |
| Male | 94 | | | 26 | 23 | | 93 | | | 0.800 | |
| Female | 15 | | | 2 | 3 | | 11 | | |  |  |
| **Age (year)^b^** | 51.3 ± 1.0 | | | 50 ± 1.9 | 47.8 ± 2.1 | | 49.9 ± 1.0 | | | 0.427 | |
| **HBeAg positive** |  | | |  |  | |  | | |  | |
| Yes | 26 | | | 7 | 9 | | 17 | | | 0.195 | |
| No | 83 | | | 21 | 17 | | 87 | | |  |  |
| **AFP (ng/ml)** |  | | |  |  | |  | | |  | |
| ≥ 400 | 72 | | | 16 | 16 | | 63 | | | 0.776 | |
| < 400 | 37 | | | 12 | 10 | | 41 | | |  |  |
| **Tumor size (cm)^b^** | 6.5 ± 0.4 | | | 5.6 ± 0.9 | 6.9 ± 0.7 | | 5.6 ± 0.3 | | | 0.156 | |
| **Tumor number (count) ^b^** 1.3 ± 0.06 | | | | 1.4 ± 0.13 | 1.3 ± 0.12 | | 1.2 ± 0.04 | | | 0.329 | |
| **Differentiation** |  | | |  |  | |  | | |  | |
| Well (I) | 0 | | | 0 | 0 | | 3 | | | 0.387 | |
| Intermediate (II-III) | 107 | | | 28 | 26 | | 101 | | |  |  |
| Poor (IV) | 2 | | | 0 | 0 | | 0 | | |  |  |
| **Tumor satellites** |  | | |  |  | |  | | |  | |
| Yes | 76 | | | 20 | 18 | | 77 | | | 0.905 | |
| No | 33 | | | 8 | 8 | | 27 | | |  |  |
| **Microvascular Invasion** | | | | | | | | | | | |
| Yes | 74 | | | 12 | 22 | | 55 | | | **0.002** | |
| No | 35 | | | 16 | 4 | | 49 | | |  |  |
| **Recurrence** |  | | |  |  | |  | | |  | |
| Yes | 80 | | | 15 | 19 | | 52 | | | **0.003** | |
| No | 29 | | | 13 | 7 | | 51 | | |  |  |
| **Expired** |  | | |  |  | |  | | |  | |
| Yes | 73 | | | 11 | 18 | | 29 | | | **< 0.001** | |
| No | 36 | | | 17 | 8 | | 75 | | |  |  |
| **Risk-free survival time (mo)^b^** | 24.8 ± 2.3 | | | 35.1 ± 4.9 | 23.2 ± 5.1 | | 42 ± 2.4 | | | **< 0.001** | |
| **Overall survival time (mo)^b^** 34.2 ± 2.3 | | | | 44.8 ± 4.3 | 31.3 ± 4.6 | | 53.5 ± 2 | | | **< 0.001** | |
| ^a^ Statistical significance was caluculated by chi-square test or fisher's exact test for categorical/binary measures and ANOVA for continuous measures.  ^b^ Data are presented as mean ± SD. | | | | | | | | | | | |

**Table S2. Clinicopathological Characteristics of HCC Subtypes Defined by OV6 and MDM2 Expression.**

| **Clinicopathological Features of HCC Subtypes Defined by OV6 and MDM2 Expression** | | | | | |
| --- | --- | --- | --- | --- | --- |
|  | **OV6/MDM2 expression** | | | |  |
|  |  | **Either high** | |  |  |
| **HCC subtypes** | **Both high (n= 68)** | **High MDM2, low OV6 (n= 69)** | **High OV6, low MDM2 (n= 38)** | **Both low (n= 92)** | **P value^a^** |
| **Sex** |  |  |  |  |  |
| Male | 60 | 60 | 31 | 85 | 0.349 |
| Female | 8 | 9 | 7 | 7 |  |
| **Age (year)^b^** | 52.3 ± 1.2 | 49.8 ± 1.2 | 47.5 ± 1.7 | 50.3 ± 1.1 | 0.128 |
| **HBeAg positive** |  |  |  |  |  |
| Yes | 21 | 12 | 8 | 18 | 0.231 |
| No | 47 | 57 | 30 | 74 |  |
| **AFP (ng/ml)** |  |  |  |  |  |
| ≥ 400 | 49 | 39 | 25 | 54 | 0.217 |
| < 400 | 19 | 30 | 13 | 38 |  |
| **Tumor size (cm)^b^** | 7.8 ± 0.5 | 5 ± 0.4 | 7.2 ± 0.5 | 5.3 ± 0.3 | **< 0.001** |
| **Tumor number (count)^b^** | 1.4 ± 0.1 | 1.2 ± 0.04 | 1.3 ± 0.09 | 1.1 ± 0.04 | **0.005** |
| **Differentiation** |  |  |  |  |  |
| Well (I) | 0 | 0 | 1 | 2 | 0.452 |
| Intermediate (II-III) | 67 | 68 | 37 | 90 |  |
| Poor (IV) | 1 | 1 | 0 | 0 |  |
| **Tumor satellites** |  |  |  |  |  |
| Yes | 48 | 48 | 29 | 66 | 0.899 |
| No | 20 | 21 | 9 | 26 |  |
| **Microvascular Invasion** | | | | | |
| Yes | 46 | 40 | 28 | 49 | 0.093 |
| No | 22 | 29 | 10 | 43 |  |
| **Recurrence** |  |  |  |  |  |
| Yes | 61 | 34 | 32 | 39 | **< 0.001** |
| No | 7 | 35 | 6 | 52 |  |
| **Expired** |  |  |  |  |  |
| Yes | 62 | 22 | 29 | 18 | **< 0.001** |
| No | 6 | 47 | 9 | 74 |  |
| **Risk-free survival time (mo)^b^** | 12.6 ± 2 | 41 ± 2.9 | 18.4 ± 3.4 | 46.4 ± 2.5 | **< 0.001** |
| **Overall survival**  **time (mo)^b^** | 20.9 ± 2.1 | 51.5 ± 2.3 | 27.1 ± 3.8 | 58.1 ± 1.6 | **< 0.001** |
| ^a^ Statistical significance was caluculated by chi-square test or fisher's exact test for categorical/binary measures and ANOVA for continuous measures.  ^b^ Data are presented as mean ±SD. | | | | | |

**Table S3. Clinicopathological Characteristics of HCC Subtypes Defined by MDM2 and CXCR4 Expression.**

| **Clinicopathological Features of HCC Subtypes Defined by MDM2 and CXCR4 Expression** | | | | | | | | | | |  | |  | |  | |  | |  | |  | |
| --- | --- | --- | --- | --- | --- | --- | --- | --- | --- | --- | --- | --- | --- | --- | --- | --- | --- | --- | --- | --- | --- | --- |
|  | | **MDM2/CXCR4 expression** | | | | | | |  | |  | |  | |  | |  | |  | |  | |
|  | |  | | **Either high** | | |  | |  | |  | |  | |  | |  | |  | |  | |
| **HCC subtypes** | | **Both high (n= 131)** | | **High MDM2, low CXCR4 (n= 6)** | | **High CXCR4, low MDM2 (n= 23)** | **Both low (n= 107)** | | **P value^a^** | |  | |  | |  | |  | |  | |  | |
| **Sex** | |  | |  | |  |  | |  | |  | |  | |  | |  | |  | |  | |
| Male | | 114 | | 6 | | 21 | 95 | | 0.926 | |  | |  | |  | |  | |  | |  | |
| Female | | 17 | | 0 | | 2 | 12 | |  |  |  | |  | |  | |  | |  | |  |  |
| **Age (year)^b^** | | 50.7 ± 0.9 | | 59.2 ± 4.7 | | 45.6 ± 1.8 | 50.4 ± 1 | | 0.021 | |  | |  | |  | |  | |  | |  | |
| **HBeAg positive** | |  | |  | |  |  | |  | |  | |  | |  | |  | |  | |  | |
| Yes | | 31 | | 2 | | 7 | 19 | | 0.357 | |  | |  | |  | |  | |  | |  | |
| No | | 100 | | 4 | | 16 | 88 | |  |  |  | |  | |  | |  | |  | |  |  |
| **AFP (ng/ml)** | |  | |  | |  |  | |  | |  | |  | |  | |  | |  | |  | |
| ≥ 400 | | 84 | | 4 | | 13 | 66 | | 0.905 | |  | |  | |  | |  | |  | |  | |
| < 400 | | 47 | | 2 | | 10 | 41 | |  |  |  | |  | |  | |  | |  | |  |  |
| **Tumor size (cm)^b^** | | 6.4 ± 0.4 | | 4.7 ± 0.6 | | 6.9 ± 0.8 | 5.6 ± 0.3 | | 0.204 | |  | |  | |  | |  | |  | |  | |
| **Tumor number (count)^b^** | | 1.3 ± 0.06 | | 1.2 ± 0.17 | | 1.2 ± 0.11 | 1.2 ± 0.04 | | 0.520 | |  | |  | |  | |  | |  | |  | |
| **Differentiation** | |  | |  | |  |  | |  | |  | |  | |  | |  | |  | |  | |
| Well (I) | | 0 | | 0 | | 0 | 3 | | 0.260 | |  | |  | |  | |  | |  | |  | |
| Intermediate (II-III) | | 129 | | 6 | | 23 | 104 | |  |  |  | |  | |  | |  | |  | |  |  |
| Poor (IV) | | 2 | | 0 | | 0 | 0 | |  |  |  | |  | |  | |  | |  | |  | |
| **Tumor satellites** | |  | |  | |  |  | |  | |  | |  | |  | |  | |  | |  | |
| Yes | | 92 | | 4 | | 17 | 78 | | 0.927 | |  | |  | |  | |  | |  | |  | |
| No | | 39 | | 2 | | 6 | 29 | |  | |  | |  | |  | |  | |  | |  |  |
| **Microvascular Invasion** | |  | |  | |  |  | |  | |  | |  | |  | |  | |  | |  | |
| Yes | | 83 | | 3 | | 18 | 59 | | 0.164 | |  | |  | |  | |  | |  | |  | |
| No | | 48 | | 3 | | 5 | 48 | |  | |  | |  | |  | |  | |  | |  |  |
| **Recurrence** | |  | |  | |  |  | |  | |  | |  | |  | |  | |  | |  | |
| Yes | | 90 | | 5 | | 15 | 56 | | 0.056 | |  | |  | |  | |  | |  | |  | |
| No | | 41 | | 1 | | 8 | 50 | |  | |  | |  | |  | |  | |  | |  |  |
| **Expired** | |  | |  | |  |  | |  | |  | |  | |  | |  | |  | |  | |
| Yes | | 81 | | 3 | | 13 | 34 | | **<0.001** | |  | |  | |  | |  | |  | |  | |
| No | | 50 | | 3 | | 10 | 73 | |  | |  | |  | |  | |  | |  | |  |  |
| **Risk-free survival time (mo)^b^** | 27.1 ± 2.2 | | 22.5 ± 9.5 | | 25.6 ± 6 | | 41 ± 2.4 | **<0.001** | |  | |  | |  | |  | |  | |  | |  |
| **Overall survival time (mo)^b^** | 35.8 ± 2 | | 47.8 ± 13.2 | | 33.5 ± 5.6 | | 52.4 ± 2 | **<0.001** | |  | |  | |  | |  | |  | |  | |  |
| ^a^ Statistical significance was caluculated by chi-square test or fisher's exact test for categorical/binary measures and ANOVA for continuous measures.  ^b^ Data are presented as mean ± SD. | | | | | | | | | | |  | |  | |  | |  | |  | |  | |

**Table S4. Summary of Clinicopathological Variables of 267 primary HCC patients.**

| **Summary of Clinicopathologic Variables** | |
| --- | --- |
| **Characteristic** | **No. of patients** |
|  | 267 |
| Sex |  |
| Male | 236 |
| Female | 31 |
| Age (year) | 22 - 73 (50) |
| HBeAg |  |
| Yes | 59 |
| No | 208 |
| AFP (ng/ml) ^a^ |  |
| ≥ 400 | 167 |
| < 400 | 100 |
| Tumor size (cm) | 1.0 - 22.0 (median, 5.0) |
| Tumor number (count) | 1 - 4 (median, 1) |
| Tumor differentiation grade | |
| Well (I) | 3 |
| Intermediate (II-III) | 264 |
| Tumor satellites |  |
| Yes | 191 |
| No | 76 |
| Microvascular invasion |  |
| Yes | 163 |
| No | 104 |
| Recurrence |  |
| Yes | 166 |
| No | 100 |
| Expired |  |
| Yes | 131 |
| No | 136 |
| Risk-free Survival time (mo) | 1 - 77 (median, 26) |
| Time of folow-up (mo) | 1 - 90 (median, 54) |
| ^a^ AFP, serum-fetoprotein. | |

**Table S5. Sequence of siRNAs targeting MDM2, CXCR4 and CXCL12 used in this study.**

| **Sequences of siRNAs targeting MDM2, CXCR4 and CXCL12** | | |
| --- | --- | --- |
| **Name** | **Forward primer** | **Reverse primer** |
| siMDM2 #1 | 5'- GGAACUUGGUAGUAGUCAAUC-3' | 5'- UUGACUACUACCAAGUUCCUG-3' |
| siMDM2 #2 | 5'- GGAUCUUGAUGCUGGUGUAAG-3' | 5'- UACACCAGCAUCAAGAUCCGG-3' |
| siMDM2 #3 | 5'- CAUAUUGUAUAUUGUUCAAAU-3' | 5'- UUGAACAAUAUACAAUAUGUU-3' |
| siCXCR4 #1 | 5'- GCCUCAAGAUCCUCUCCAAAG-3' | 5'- UUGGAGAGGAUCUUGAGGCUG-3' |
| siCXCR4 #2 | 5'-GCUGUUUAUGCAUAGAUAAUC -3' | 5'- UUAUCUAUGCAUAAACAGCUG -3' |
| siCXCR4 #3 | 5'- CCUGGAAAUCAUCAAGCAAGG -3' | 5'- UUGCUUGAUGAUUUCCAGGAG -3' |
| siCXCL12 #1 | 5'- GAAGAACAACAACAGACAAGU -3' | 5'- UUGUCUGUUGUUGUUCUUCAG -3' |
| siCXCL12 #2 | 5'- CGAAGCUAAAGUGGAUUCAGG -3' | 5'- UGAAUCCACUUUAGCUUCGGG -3' |
| siCXCL12 #3 | 5'- GGCAAUUUGUAAAGAAAUAUA -3' | 5'- UAUUUCUUUACAAAUUGCCAG -3' |
| siβ-Catenin #1 | 5'- GAUGGUGUCUGCUAUUGUACG -3' | 5'- UACAAUAGCAGACACCAUCUG -3' |
| siβ-Catenin #2 | 5'- GGACAAGGAAGCUGCAGAAGC -3' | 5'- UUCUGCAGCUUCCUUGUCCUG -3' |
| siβ-Catenin #3 | 5'- GAAUACAAAUGAUGUAGAAAC -3' | 5'- UUCUACAUCAUUUGUAUUCUG -3' |

**Table S6. Sequence of PCR primers used in this study.**

| **Sequence of PCR Primers Used in This Study** | | |
| --- | --- | --- |
| **Gene** | **Forward primer** | **Reverse primer** |
| CD133 | 5'-GCCACCGCTCTAGATAC  TGC-3' | 5'-TGTTGTGATGGGCTTGT  CAT-3' |
| EpCAM | 5'-CGAGTGAGAACCTACTGGA  TCA-3' | 5'-CCATTTACTGTCAGGTCC  ATT-3' |
| CD90 | 5'-CTAGTGGACCAGAGCCT  TCG-3' | 5'-GCACGTGCTTCTTTGTC  TCA-3' |
| ABCG2 | 5'-CAGGTTACGTGGTACAAGA  TGA-3' | 5'-GATCAGTGATAAGCTCCAT  TCC-3' |
| CD24 | 5'-TGAAGAACATGTGAGAGGT  TTGAC-3' | 5'-GAAAACTGAATCTCCATTC  CACAA-3' |
| Sox2 | 5'-CAAGATGCACAACTCGG  AGA-3' | 5'-GCTTAGCCTCGTCGATG  AAC-3' |
| Nanog | 5'-CTGCTGGACTGAGCTGGTT  GCC-3' | 5'-GCTGAGGCCTTCTGCGTC  ACA-3' |
| Oct4 | 5'-AGTGAGAGGCAACCTGG  AGA-3' | 5'-ACACTCGGACCACATCC  TTC-3' |
| Bmil | 5'-AGAGATCGGGGCGAGAC  AAT-3' | 5'-TTGCTGGTCTCCAGGTA  ACG-3' |
| MDM2 | 5'-TCTGAAAGCACCAGCACTTG-3' | 5'-TACTGAACACGCCTCCC  ATC-3'' |
| CXCR4 | 5'-TACACCGAGGAAATGGG  CTCA-3' | 5'- AGATGATGGAGTAGATGG  TGGG-3' |
|  |  |  |
| CXCL12 | 5'- TCAGCCTGAGCTACAGATGC-3' | 5'-CTTTAGCTTCGGGTCAATGC<3' |
| HBx | 5'-TGCTGCCAACTGGATCCTG5'-3' | 5'-ATGCCTCAAGGTCGGTCGT-3' |
| β-catenin | 5'- GGCCCAGAATGCAGTTCGCCTT-3' | 5'- AATGGCACCCTGCTCACGCA-3' |
| Actin | 5'-CCCTGGCACCCAGCAC-3' | 5'-GCCGATCCACACGGAG-3' |

**Supplementary Figures**


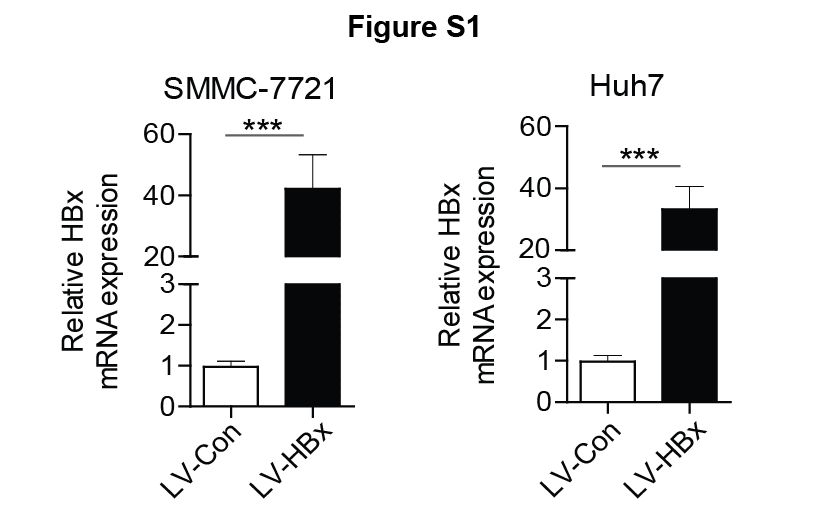


**Supplementary Figure 1.** Expression of exogenous HBx in SMMC-7721 and Huh7 cells transfected with LV-Con or LV-HBx were detected by qRT-PCR. The fold change was determined using the delta-delta Ct method. Quantified mRNA levels were normalized to β-actin and presented relative to the controls. Data represent Mean ± SD.


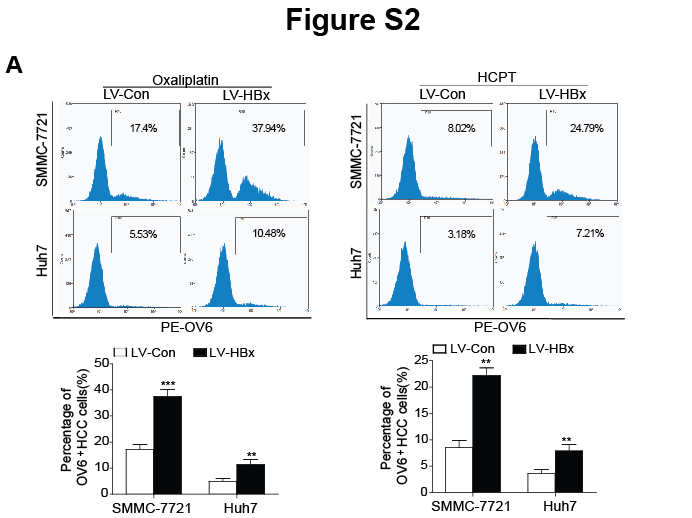


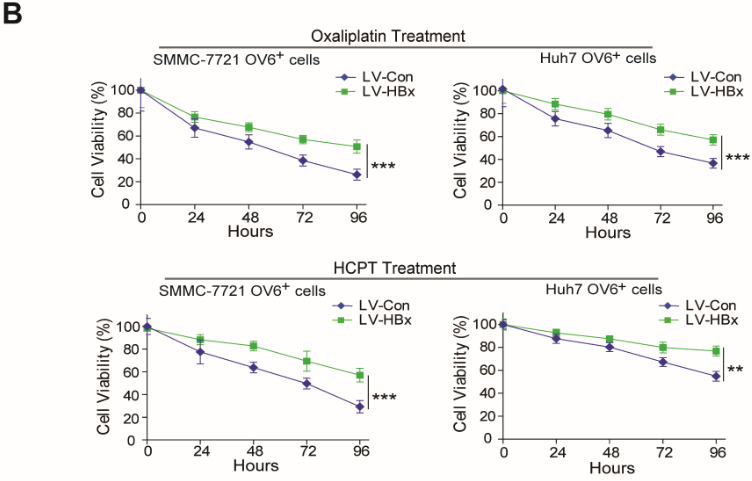


**Supplementary Figure 2.** (A) SMMC-7721 and Huh7 cells were infected with LV-HBx or LV-Con ant then treated with 5 μM Oxaliplatin or 10 μM HCPT for 4 days. The proportion of OV6^+^ cells were detected via flow cytometry. Representative results from 3 independent experiments are shown. (B) OV6^+^ HCC cells were treated with 5 μM Oxaliplatin or 10 μM HCPT and cell viabilities were measured by CCK-8 assay at indicated time points. Experiments were performed in triplicate and all data are shown as Mean ± SD. **p<0.01 and ***p<0.001.


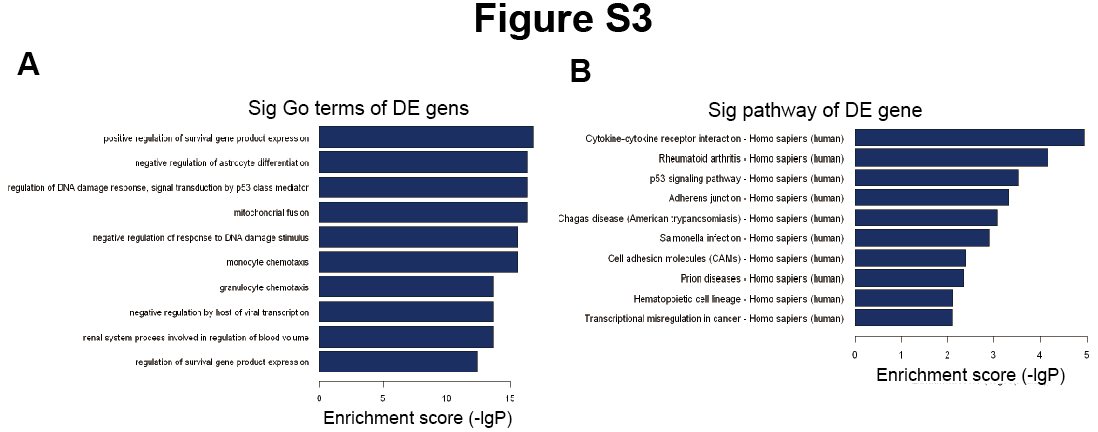


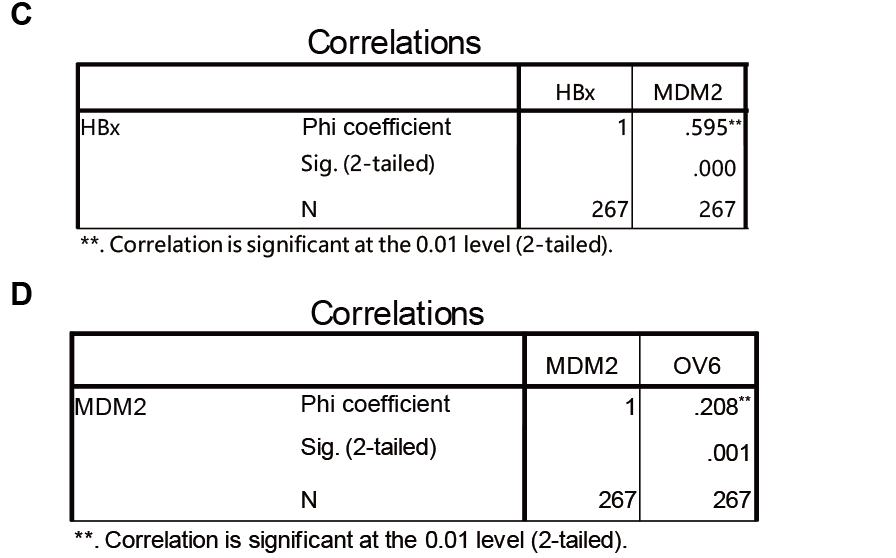


**Supplementary Figure 3.** (A) GO analysis associating differentially expressed mRNAs with GO categories. Significant GO terms influenced by overexpression HBx in OV6^+^ HCC cells. The vertical axis represents the GO category, and the horizontal axis is the log of the P value (-LgP). (B) Signaling pathway analysis for differentially expressed mRNAs in OV6^+^ HCC cells expressing vector or HBx. The vertical axis is the pathway category, and the horizontal axis represent the log of the P value (-LgP). (C) Correlation analysis of HBx and MDM2 expression in HCC samples (Phi coefficient = 0.595; P < 0.0001). (D) Correlation analysis of MDM2 and OV6 expression in HCC samples (Phi coefficient = 0.208; P = 0.001). In C and D, the correlation is significant at the 0.01 level (2-tailed).


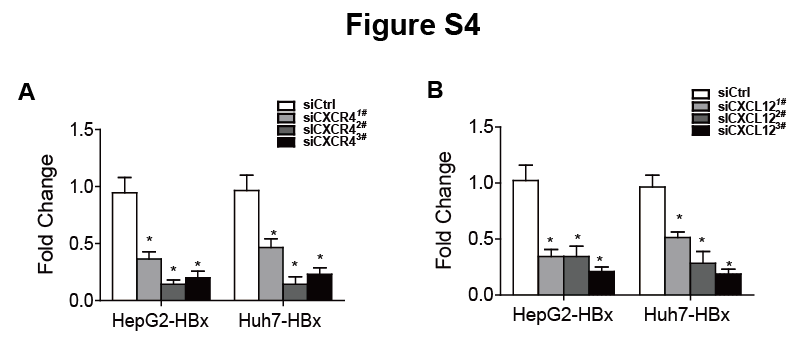


**Supplementary Figure 4.** HCC cells (HepG2, Huh7) that stably expressed HBx were transfected with siRNAs targeting negative control or CXCR4 or CXCL12 (3 sequences: #1, #2, #3). Then OV6^+^ CSCs from above cells were sorted and the knockdown efficiency of CXCR4 (A) or CXCL12 (B) in mRNA expression levels was detected by qRT-PCR, respectively.


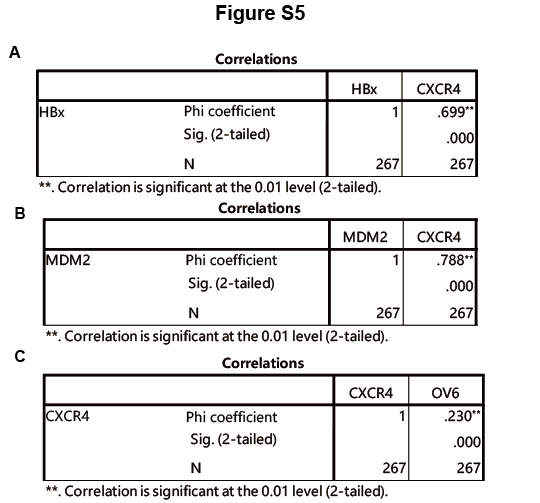


**Supplementary Figure 5.** HBx, MDM2 and CXCR4 expression were examined in tumor specimens from 267 patients with HBV-related HCC, Positive correlations between HBx and CXCR4 (A), MDM2 and CXCR4 (B), and CXCR4 and OV6 (C) were observed. (p<0.001)
